# What women want: qualitative analysis of consumer evaluations of maternity care in Queensland, Australia

**DOI:** 10.1186/s12884-014-0366-2

**Published:** 2014-10-26

**Authors:** Loretta C McKinnon, Samantha J Prosser, Yvette D Miller

**Affiliations:** School of Public Health and Social Work, Institute of Health and Biomedical Innovation, Queensland University of Technology, Kelvin Grove, 4059 QLD Australia; Queensland Centre for Mothers & Babies, School of Psychology, The University of Queensland, Queensland, Australia

**Keywords:** Hospitals, Birth, Consumer participation, Midwifery, Postnatal care, Evaluation, Service improvement, Reform

## Abstract

**Background:**

Maternity care reform plans have been proposed at state and national levels in Australia, but the extent to which these respond to maternity care consumers’ expressed needs is unclear. This study examines open-text survey comments to identify women’s unmet needs and priorities for maternity care. It is then considered whether these needs and priorities are addressed in current reform plans.

**Methods:**

Women who had a live single or multiple birth in Queensland, Australia, in 2010 (*n* 3,635) were invited to complete a retrospective self-report survey. In addition to questions about clinical and interpersonal maternity care experiences from pregnancy to postpartum, women were asked an open-ended question “*Is there anything else you’d like to tell us about having your baby?”* This paper describes a detailed thematic analysis of open-ended responses from a random selection of 150 women (10% of 1,510 who responded to the question).

**Results:**

Four broad themes emerged relevant to improving women’s experiences of maternity care: quality of care (interpersonal and technical); access to choices and involvement in decision-making; unmet information needs; and dissatisfaction with the care environment. Some of these topics are reflected in current reform goals, while others provide evidence of the need for further reforms.

**Conclusions:**

The findings reinforce the importance of some existing maternity reform objectives, and describe how these might best be met. Findings affirm the importance of information provision to enable informed choices; a goal of Queensland and national reform agendas. Improvement opportunities not currently specified in reform agendas were also identified, including the quality of interpersonal relationships between women and staff, particular unmet information needs (e.g., breastfeeding), and concerns regarding the care environment (e.g., crowding and long waiting times).

## Background

Internationally, researchers and policy makers have critiqued the features of a high quality maternity system that is aligned with the values of the community that it serves [1-4]. Objectives for maternity care reform that have arisen from such enquiry include improved: monitoring (e.g. the ongoing assessment of workforce needs) [2], safety [5], equity in access [2], regulated education and training for the maternity care workforce [6], communication and co-operation between providers [2], and improved opportunities for consumer decision-making and choice [2-4].

Women’s maternity care experiences in Queensland, Australia, are influenced by both state and national policy and legislation, and reforms have been proposed at both levels. The Maternity and Newborn Services in Queensland Work Plan (2008–2012) [7] was devised in 2004 and The National Maternity Services Plan 2010 [8] in 2010. Some aspects of these reform documents have been implemented while others are ongoing. Combined, the reform documents focus on seven goals. Five goals are common to both state and national reform. These are enhancement of: consumer involvement and choice, equity, service delivery, workforce and postnatal care. Two goals (enhanced continuity of care and infrastructure), only feature as discrete goals in the national reform plan (summarised in Table 1).

**Table 1:** **Summary of objectives for maternity care reform expressed in national and state-level documentation**

**Reform objectives**	**National** ^**a**^	**Queensland** ^**b**^
*Consumer involvement and choice:* ensuring that care is women-centred, that women are informed and have access to choices (e.g. access to different models of care).	“Access” Women-centred care, reflecting the needs of each woman. Make care available in a range of settings. Ensure women have access to information	Consumer involvement and choice
*Equity*: improving outcomes for Aboriginal and Torres Strait Islander women and women in remote and rural areas, including provision of culturally competent staff and the availability of local maternity care for rural and remote dwelling women	Culturally competent care in a range of settings close to where they live to contribute to closing the gap between the health outcomes of Aboriginal and Torres Strait Islander people and non-Indigenous Australians	Improve outcomes for Aboriginal and Torres Strait Islander peoples
	Care sensitive to all potentially vulnerable groups, e.g. those with medical, socioeconomic or other risk factors that may increase the likelihood of poor outcomes	Improve care in rural and remote areas of Queensland
	Women in rural and remote Australia have increased access to local maternity care	
*Service delivery*: provision of high-quality, safe, evidence-based care that is sustainable	Safe and sustainable quality system	Quality and safety of care
	High quality, evidence-based care	Integration of care across settings
*Workforce*: resourcing a workforce that is qualified to provide woman-centred care that is clinically safe and based on a wellness paradigm	Appropriately trained and qualified maternity health professionals	Sustainability of the maternity care workforce
	Support rural and remote and Aboriginal and Torres Strait Islander workforce. Facilitating interdisciplinary collaboration	
*Postnatal care*	Increased access to midwifery postnatal care, outside hospital settings, for at least two weeks after birth	Improve care in the postnatal period
*Continuity of care*	Continuous maternity care able to be provided to all women	
*Infrastructure*: care should be provided within a safe, high-quality system. Planning and design of maternity services should be woman-centred.	Planning and delivery of maternity care should be consistent with meeting the goals outlined above including providing high quality, women-centred care by a sustainable workforce.	

^a^Derived from the National Maternity Services Plan: 2010 [8]. ^b^Derived from the Maternity and Newborn Services in Queensland Work Plan 2008–2012 [7].

The proposed reforms offer logical and positive directions for improving maternity services, but determining their relevance and importance for current maternity care consumers is essential for ensuring that arising policy and research agendas are woman-centred [9]. Over the past two decades, studies and advisory reports [4,10-12] have used both quantitative and qualitative methods [13] to explore discrete components of maternity care (e.g. antenatal or postnatal care [14]). This study used qualitative data from recent maternity care consumers in Queensland, Australia, concerning their perceptions of care throughout the maternity [9] period (from pregnancy to postnatal care), to identify women’s priorities for maternity care and consider their alignment with state and national maternity care reform objectives.

## Methods

### Study design

The Having a Baby in Queensland Survey 2010 was a cross-sectional, retrospective evaluation of women’s experiences of maternity care in Queensland, Australia [15]. Women who experienced a live singleton or multiple birth in Queensland between April and May 2010 were invited to confidentially participate by the Queensland Registry of Births, Deaths and Marriages, using hospital notifications and birth registrations. Personal details of the invited sample were never released to the researchers. Women were contacted by mail approximately 4 months after birth and invited to complete the survey by mail, online or via a telephone interview.

The survey assessed care during pregnancy, labour and birth, and after birth, along with infant and maternal characteristics. The survey was pilot tested and revised in 2009 [16]. The current study analysed responses to the final open-ended question, ‘*Is there anything else you’d like to tell us about having your baby?*’ A general inductive approach was used to analyse the qualitative data collected as it provides a pragmatic and useful means of addressing focussed evaluation questions such those of interest in this study [17].

### Sample

Of the 10,019 eligible women who received a survey package, 3,651 returned usable surveys (response rate = 36.4%). Women who completed the telephone survey (*n* 16) were excluded due to incomplete data. Of the remaining 3,635 women, 1,510 (41.5%) responded to the final open-ended question. This study considered a random sample of approximately 10% of these women (*n* 150), with all 1,510 respondents having equal likelihood of being selected. Characteristics of the study sample were compared with: all women who completed the open-text survey item (*n* 1,510), all women who completed the survey^a^ (*n* 3,635), and the 2010 Queensland birthing population (*n* 61,027; see Table 2) [18].

**Table 2:** **Socio-demographic and birthing profile by sample**

**Characteristic**	**Having a Baby in Queensland 2010 sample**						**Queensland birthing population, 2010**	
	***Respondents assessed in this study***		***Respondents completing the open-ended item***		***Total respondents***			
	***(n = 150)***		***(n = 1,510)***		***(n = 3,635)***		**(** ***n*** **= 61,027)** ^**a**^	
	**n**	**%**	**n**	**%**	**n**	**%**	**n**	**%**
Maternal age (years)								
< 20	2	1.3	23	1.5	80	2.2	3,344	5.5
20-24	17	11.3	160	10.6	396	10.9	10,616	17.4
25-29	39	26.0	403	26.7	951	26.2	17,314	28.4
30-34	49	32.7	476	31.5	1,168	32.1	17,607	28.9
35-39	26	17.3	288	19.1	664	18.3	10,037	16.5
≥ 40	8	5.3	69	4.6	137	3.8	2,109	3.5
* Missing*	*9*		*91*		*239*		*0*	
Education^b^								
No formal qualifications	2	1.3	20	1.3	57	1.6	-	-
Year 10 or 12 equivalent	31	20.7	369	24.4	984	27.1	-	-
Trade/apprenticeship, certificate/diploma	58	38.7	452	29.9	1,086	29.9	-	-
Bachelor degree or higher	59	39.3	666	44.1	1,486	40.9	-	-
* Missing*	*0*		*3*		*22*		-	-
Area of residence								
Major city	98	65.3	969	64.2	2,281	62.8	36,263	59.4
Regional (inner and outer)	48	32.0	473	31.4	1,201	33.1	22,241	36.4
Remote and very remote	2	1.3	37	2.5	82	2.3	1,921	3.1
Outside Queensland	0	0	14	0.9	30	0.8	602	1.0
* Missing*	*2*		*17*		*41*		*0*	
Mode of birth								
Unassisted vaginal	82	54.7	834	55.2	2,026	55.7	35,077	57.5
Assisted vaginal	18	12.0	185	12.3	420	11.6	5,892	9.7
Caesarean section	49	32.7	487	32.3	1,172	32.2	20,058	32.9
* Missing*	*1*		*4*		*17*		*0*	
Parity								
Primipara	70	46.7	731	48.4	1,646	45.3	24,878	40.8
Multipara	80	53.3	777	51.5	1,969	54.2	36,149	59.2
* Missing*	*0*		*2*		*20*		*0*	
Place of birth								
Hospital (public)	96	64.0	863	57.2	2,043	56.2	41,509	68.0
Hospital (private)	45	30.0	576	38.1	1,466	40.3	18,344	30.1
Birth centre	4	2.7	41	2.7	72	2.0	674	1.1
Home birth	3	2.0	23	1.5	28	0.8	85	0.1
Other	2	1.3	5	0.3	12	0.3	415	0.7
* Missing*	*0*		*2*		*15*		*0*	

^a^Figures from AIHW 2010 [18]. ^b^Highest level of education. Maternal education was not reported by AIHW in 2010.

The study sample (*n* 150*)* was characteristically similar to the overall survey sample and the subset of the sample that completed the open-text item. Compared to the overall survey sample, the study sample did not differ in terms of age, education level, area of residence, mode of birth, or parity, but appeared more likely to have given birth in a public hospital (see Table 2). The study sample also appeared characteristically similar to the total population of women birthing in Queensland in 2010 (*n* 61,027); [18] the majority of women were aged between 25 and 39 years, were multiparous, and gave birth in public hospitals. A slightly lower proportion of women in the current study had an unassisted vaginal birth compared to the overall Queensland birthing population (54.7% compared to 57.5%); however, this was the most common mode of birth in both samples. Women in the current sample appeared more likely to be urban dwellers (65.3% compared to 59.4%), less likely to be multiparous (53.3% compared to 59.2%), less likely to be aged <20 (1.3% compared to 5.5%) and less likely to be aged 20–24 (11.3% compared to 17.4%) compared to the overall Queensland birthing population.

### Ethical approval

Ethical approval for The Having a Baby in Queensland Survey, 2010 and subsequent analyses was obtained from The University of Queensland Behavioural & Social Sciences Ethical Review Committee on 1^st^ June, 2010 (Clearance #2010000613).

### Analysis

A general inductive analysis [19,20] was conducted comprising several steps. The first was data familiarisation, in which the first author read and re-read transcripts to become accustomed to the data. After familiarisation, short phrases or ‘codes’ were assigned to data to reflect meaning based on identified concepts, topics, ideas or phrases. The purpose of the analysis was to identify specific topics and concerns raised by women that were pertinent to maternity care improvement. Attention was paid to when women’s perspectives converged and when they differed, and statements and quotes with similar meanings were highlighted and grouped together. The identification of patterns in the generated ‘codes’ allowed themes relevant to maternity care provision to be developed.

The constant comparative approach was applied whereby codes and themes were continuously developed and revised based on re-reading of women’s responses and consideration of previous coding [21]. Although a formal second coding was not undertaken, the co-authors reviewed much of the raw data to refine and add codes and themes. Reflection, discussion and revision of themes using the raw data occurred during fortnightly meetings of the research team (including all authors) to discuss discrepancies until consensus was achieved. This process was purposeful in terms of identifying opportunities for maternity care improvement from women’s comments. However, the derivation of themes was data-driven rather than being focussed on collecting evidence relevant to a particular theory or model. Counts of themes were undertaken and the themes presented here represent those most commonly raised by women.

## Results

The open-text question generated a wide range of responses*,* as is typical for this type of data collection [22]. Response length ranged from no response through to several paragraphs. Women were not restricted with regard to the amount they could write, or the length of time they could speak if participating in a phone interview. Approximately one-third of women (*n* 52) expressed satisfaction with at least some aspect of their care, while two-thirds of women (*n* 95) highlighted at least one aspect of care requiring improvement (some in addition to positive comments). While the presented results focus on themes representing the most popular ‘calls’ for improvement, positive comments relevant to each theme are also provided to enhance understanding of how care may be improved.

Four main themes emerged relevant to improving women’s experiences of maternity care: quality of care, access to choices and involvement in decision-making, unmet information needs, and concerns about the care environment. The first two themes were the most commonly expressed, each being noted by approximately one-third of women (*n* 55 and *n* 46, respectively). The next most commonly noted themes were unmet information needs (17%, *n* 26) and concerns regarding the care environment (10%, *n* 16).

### Quality of care

Concerns regarding the quality of care included interpersonal concerns, disregard of information provided by women (and in their medical records), and issues attributed to low staff numbers. Although few women expressed dissatisfaction with the technical expertise of staff, when mentioned this was reported to cause considerable physical and/or emotional distress.

Women generally referred to midwives or nurses in their comments, with fewer references to doctors, obstetricians, or lactation consultants. Inconsistency in the service received across individual midwives or nurses was common. Women expressed satisfaction and gratitude for the service provided by some staff alongside extreme disappointment in the service provided by others. Below is a quote exemplary of respondents’ contrasting sentiments concerning the service provided by individual staff members.

Respondent 35: *“There was only one midwife who continually chopped and changed her hour-to-hour care of my baby…I was very angry with her, because she treated me like an idiot and refused to let me hold my baby because ‘it is too draining for him’…Having said that, the other midwives were considerate and helpful”.*

#### Interpersonal concerns

A number of women reported that staff had been rude to them, their support people, or family members. Some women commented that hospital staff were only concerned with the welfare of the baby, and showed little concern for their wellbeing. Women emphasised that their birthing experience was enhanced by caring, understanding and empathetic staff that ensured they were well informed.

Respondent 60: *“During the time before, during and after the birth the staff were more than caring and informative. They explained all details clearly and thoroughly”.*

Specific staff behaviours conveying genuine care included taking women’s concerns seriously and maintaining eye contact. Uncaring behaviours included rolling of eyes, laughing, being spoken about in a negative way within hearing distance, and the exertion of physical force towards mother or baby. Some women reported feeling pressured, judged and discriminated against by medical staff for their decisions or preferences, particularly in relation to decisions about place of birth (home or hospital), mode of birth (vaginal or caesarean section), refusal of medical intervention, and infant feeding (breast milk or infant formula).

Respondent 48: *“… there was a doctor and a midwife who disagreed with my decisions (to have a vaginal birth following a caesarean, at home) and made it very known. They said I was neglectful and said they would report me to the Department of Child Services. This sent me into a whirlwind of deep depression, as I felt so misunderstood… The doctor made me feel as though I had harmed the baby (he had to be resuscitated at birth). It took only a month or so to get over, but at the time the only thing stopping me from killing myself was that I knew my children needed me”.*

Respondent 64: *“When I found out my baby was breech, I was given the option to have the baby turned, but declined. I was asked by a couple of midwives why I chose this as turning was a common safe option. I felt a little pressured into trying to have a vaginal birth”.*

Respondent 47: *“I explained what we had been taught and said that was how I wanted to do it* [breastfeeding]. *But she said it was ‘rubbish’ and laughed about it with the second midwife in the room”.*

Women spoke of midwives as strict gatekeepers to the medical care that they and their babies received. The quotes below exemplify women’s experiences of having pain relief withheld or being forced to birth in positions that caused pain or discomfort, sometimes described as triggers for lasting emotional or physical trauma.

Respondent 58: *“I begged her* [midwife] *for pain relief, which she wouldn’t give me even though there was plenty of time (8 hours)… I was screaming for help and I begged for a doctor, which she refused to get. I would never go back to {Facility Name} to have a baby again for fear of my own life and my child’s life. I constantly have flash backs and nightmares about my birth. I hope to one day be able to get past this fear of childbirth and to have another baby. I will not be going back to {Facility Name}, I will go private if I ever get over my trauma!!!”.*

Respondent 101: *“…Was also not happy that the midwife forced me to lay on the bed on my back all the time. It caused incredible discomfort (which is also what I had said I wanted to avoid in my birth plan)…”.*

#### Failure to respond to information

Staff failed to acknowledge information that women provided or concerns that they expressed, frequently articulated as staff “*not listening*”. In particular, women were frustrated at “*not being believed*” regarding their reported stage of labour. Noted consequences were that some women did not have time to receive any or adequate pain relief, and partners or support people missed being involved in the birth or were prevented from supporting the mother as planned. Several respondents reported that medical staff ignored them when they expressed concern that their labour was not progressing normally.

Respondent 36: *“It took several hours during the labour for me to be listened to when I was insisting there was something wrong with my labour. Injury to my baby could have been avoided”.*

Women expressed concern about the danger of staff disregarding information provided by patients or medical records. Some women described physical and emotional stress endured either by themselves or their babies as a result.

Respondent 62: *“…my baby had a respiratory infection from the meconium he’d been inhaling. Had the doctor listened to me in the first place my birth would have been very different and maybe I wouldn’t have had the emotional issues I had during the last week of pregnancy and first couple of weeks after childbirth”.*

#### Inadequate staff availability

Many women reported that their care suffered due to staff being busy or inadequate staff numbers. Several of the interpersonal factors already raised, such as not being “*listened to*” or medical records being dismissed, were attributed to insufficient staff numbers.

Respondent 38: *“By the time I was at the end of pre labour and back up at the hospital I felt that I wasn’t being listened to because they were busy. That they were rushing me and not paying much attention to me and my needs. As things went on I felt very scared and unsure of what is going on”.*

Women described being left alone, and feeling afraid or nervous by the limited contact with midwives or nurses. Other perceived consequences of staff shortages were not receiving pain relief or other medication (or receiving it at the incorrect time), long delays for assistance, and not receiving the practical help that they needed.

Respondent 117: *“My medication was not given at the correct times as the midwives were ‘too busy’. I was not helped with the baby which made me feel afraid and nervous”.*

Respondent 78: *“One night I was on my own and unwell and I buzzed for the nurse, it took 1.5 hours for a nurse to see me. Apparently there was only 1 nurse for every 20 babies!”.*

#### Staff technical expertise

Some women spoke of staff errors in issuing medications or identifying and appropriately responding to medical situations, resulting in perceived additional risk and in some cases, actual physical trauma to mother or baby. A number of women felt that the physical trauma that they endured may have been preventable.

Respondent 117: *“…I was transported to theatre for an emergency caesarean. Once full epidural block done and I was on the table, the OB/GYN checked and I was fully dilated and so they ripped the baby out with forceps, leaving me with a 4th degree tear …”.*

Respondent 101: *“Was not at all happy that I was made to push for an hour when there was still cervix in the way. It caused permanent damage to my abdominal muscles and could have been avoided had the midwife just checked me out”.*

### Access to choices and involvement in decision-making

Women indicated that their involvement in decision-making about their care had been compromised (e.g. in relation to the model of care available or preferred mode of birth) and/or described the difficulties in obtaining their preferred maternity care options or outcomes. Other women commented more broadly on the importance of having choices in relation to their maternity care.

#### Model of care

Women’s model of care enhanced or detracted from their birthing experience. When women commented on a model of care that they wanted to have available, or wanted to be promoted to other women, it was most commonly midwifery-led care, specifically birth centre care. These models were valued because it was felt that they increased the likelihood of having a natural birth (usually described as vaginal and drug-free) due to the relaxed nature of the environment, capacity for continuity of care, and opportunities to develop a rapport with maternity care staff.

Respondent 6: *“All women should be able to access Birth Centre model of care - it is far superior to the hospital as you build a relationship with midwives and you can call them at any time with concerns* etc. *…”.*

Some women expressed that their care experience was enhanced by seeing the same person throughout the maternity period (i.e., having continuity of carer). Other women felt that not having access to continuity of care had a negative impact on their overall satisfaction. Continuity of carer was expressed as desirable across the duration of the hospital stay, throughout the maternity period as a whole, and sometimes even across pregnancies.

Respondent 140: *“The policy of rotating staff around the hospital needs review. While I understand the need for staff to continually learn new skills, it was very unsettling to have a new nurse every shift in the maternity ward. In five days I had the same nurse only once, all others were new every shift”.*

Respondent 95: *“… it would be nice to meet with one midwife right through the pregnancy/labour/birth for a more comfortable experience”.*

#### Preferences for labour and birth

In planning their labour and birth, a number of women commented on the importance of access to particular options, such as water immersion or vaginal birth after caesarean section (VBAC). As already discussed, some women described how aspects of the care they received interfered with their preferences for labour and birth or limited the choices available to them (e.g. pain relief). Women talked about not having the options or preferences for birthing that they had anticipated, even in cases where this had been discussed with maternity carers prior to labour.

Respondent 22: *“…The facilities were great, but during the birth I was left alone with my husband and when I asked for help I was only offered gas when I wanted ‘support and encouragement’ , which we discussed at my antenatal appointments”.*

Respondent 101: *“Was led to believe during appointments that I would have more control over the birth of my baby than I had”.*

#### Infant care decisions

Many women felt that their choices for infant feeding were restricted. In addition, some women spoke of procedures performed on their babies after birth that were not approved by either themselves or their partner.

Respondent 85: *“.... I was discriminated against in the hospital because my baby was bottle fed. I was not allowed to feed her out of my room or have the formula in the vision of other mums. I think this is disgusting”.*

Respondent 42: *“When my child was in neonatal care, she was given the drip, medicine, x-ray and medically examined without consulting with us…”.*

#### Limited choices for local maternity care

Some women indicated that they were limited in terms of the type of birth available to them or postnatal care options available to them locally. This was not limited to women living in remote areas.

Respondent 91 (Major city): *“…I did a lot of research and preparation for my birth. There was not a hospital within 2 hours driving distance which catered for water birth…”.*

Respondent 127 (Regional): *“…. We were offered post natal care for our babies in our town up to the age of 8 weeks after that we were told we had to go to a child health nurse, which is not available to us without travelling. I found that I needed more help with breastfeeding and sleep after my baby was 2 months old and with another child at school travelling for me wasn’t ideal. … the post natal nurse travelled up to us at our local hospital once a week. Why couldn’t a nurse do the same for babies over 8 weeks?”.*

#### Assertiveness, cost and choices

Women who were successful in obtaining their preferred choices for aspects of their maternity care described their own assertiveness or financial commitment as essential for obtaining their preferences. Some women felt that it was only through being forceful, demanding or determined that they were able to receive care that met their needs.

Respondent 38: *“…My husband almost missed the birth of our son. They were following text book and I feel that, this needs to change. If it wasn’t for me being so demanding, I believe I would of had my baby alone…”.*

Women who were successful in having their preferences met for model of care, vaginal birth after caesarean, or infant feeding often noted significant personal financial outlay to facilitate this.

Respondent 91: *“We chose to hire an independent midwife to achieve a calm water birth with a known medical professional (midwife) who was guaranteed to attend the birth of our baby. We paid in excess of $5000 privately (no rebates available) and it was worth every cent for the quality of care we received in the comfort of our own home…”.*

Respondent 79: *“We experienced problems breastfeeding and spent considerable money on a lactation consultant once we got home, and it was money well spent as we are still breastfeeding now”.*

### Unmet information needs

Respondents expressed a number of unmet information needs during the antenatal, intrapartum and postnatal periods. The most common problems reported by women were receiving inconsistent information from hospital staff, not having adequate information when they returned home with their baby, and specifically, not having sufficient information regarding breastfeeding either during or after their hospital stay.

#### Inconsistent or insufficient information provided by hospital staff

Women reported receiving conflicting information from different midwives within the same facility, and sometimes from the same midwife. Women noted that these situations caused them considerable stress, anxiety and frustration.

Respondent 47: *“My antenatal classes were done at the same hospital, yet the midwives contradicted each other. I was extremely unhappy with this”.*

Respondent 109: *“Very disappointed with the midwifery care post birth in the maternity ward. Most of the midwives had different opinions and gave different information to the last. One care facility should have their staff all teaching the same education”.*

A number of women reported that they did not receive adequate information on how to care for their baby while in hospital.

Respondent 100: *“Was not given support after having the baby. Midwives would only come to check baby’s temperature and that is all. Had to figure the rest out all by myself”.*

Respondent 76: *“I found the advice in maternity ward contradictive and inconsistent and I felt ill informed with what was happening to my baby, what I should be doing”.*

Others commented that there was comparatively more information available regarding the birthing process and time in hospital, yet insufficient information provided about how to care for the baby once they returned home.

Respondent 81: *“We have lots of support by care providers during pregnancy and birth, but it all stops after birth and discharge…”.*

In addition to practical issues surrounding care for the baby, women wanted to know about relevant community organisations and parenting groups.

Respondent 11: *“Would like it to be easier to find out about free services/groups/activities for mum & bubs in my area. I only seem to be able to find out this information through friends (word of mouth)”.*

Women described logistic and financial barriers to attending postnatal appointments to obtain the information and care they needed. Difficulties such as caring for other children, recovering from a caesarean section and being single parents were all noted. Preferences were expressed for either receiving information in hospital or through home visits. Some women noted that they would have been prepared to drop in to a community centre to receive postnatal information, but such services were not provided in their local area. Some women suggested that classes regarding postnatal care would be a useful way for their information needs to be met.

Respondent 9: *“Would like to see a class attached to offered as an extra to sign up for, for the first three months, how to care for your baby all little things that may be helpful as a first time mum”.*

Respondent 5: *“You attend antenatal classes for six weeks that focus on giving birth and breastfeeding only. Then when you actually have a baby at home you are given no information. Why focus on the birth when at the end of the day its 1 day out of your life and there will be someone giving you the info you need at the time and assisting you. Why not give classes on the basics in care of your newborn…”.*

#### Inadequate breastfeeding information

Inadequate information regarding breastfeeding was a common concern. Some mothers described relying heavily on the Australian Breastfeeding Association or lactation consultants due to a lack of breastfeeding support in hospital. Those who used lactation consultants generally found them helpful, yet multiple women raised concerns regarding the availability and cost of this assistance.

Respondent 149: *“Teaching mother and baby to breastfeed successfully should be made easier and either government funded or much cheaper”.*

#### Other unmet information needs

Some women noted that their choices were limited by having minimal information on the models of care available to them.

Respondent 95: *“More information regarding your choices of care during pregnancy (I have had 2 children now, still not sure)”.*

Women in rural and remote areas reported unique unmet information needs. In particular, these mothers reported that they felt that they did not receive enough information regarding where they would birth, when they should attend hospital, or access to postnatal care after discharge. Several women described this lack of information as exacerbating their stress at an already challenging time. Multiparous women were another group who expressed unique concerns about unmet information needs. Those who commented felt that important information was withheld from them on the assumption that they should already know what to do.

Respondent 133: *“This being my second child, I felt that the nurses had the assumption that I knew what I was doing in regards to breastfeeding and setting up a routine. I felt they stayed away, especially of an afternoon and evening”.*

### Concerns about the care environment

Some women expressed issues regarding aspects of the environment at their antenatal care service or birth facility. Most commonly noted was the limited capacity for partners to stay or visit, crowding in the birthing and postnatal rooms, and long waiting times for antenatal appointments. Additionally, women were disappointed that there was nowhere to stay for a longer period with their baby for additional support, and dissatisfaction with the food supplied and hospital cleanliness.

Women were dissatisfied that there were no facilities for partners to rest or stay at the hospital or birth centre or that partners were not allowed to visit when they wanted to.

Respondent 99: *“Was not happy that my husband was unable to stay with me all day and night if he wanted, was made to leave… He wanted to stay with me after a traumatic birth and first few days and was not allowed. I needed him there”.*

However, other women suggested that visiting hours and the number and type of visitors allowed should be more strongly enforced, highlighting a need to balance differences in women’s preferences.

Respondent 106: *“Visitors should be restricted to 4 maximum as on the ward I was on, one mother had at least 6–10 people at a time. This was extremely loud and I had to leave the ward with my baby and visitors in order to be heard. The midwives/nurses tried to tell them but it did not work. A policy needs to be adopted to try and prevent this problem/disruption for the other mothers”.*

Some women indicated that they would have liked to have access to a facility where they could stay for longer after birth or after hospital discharge.

Respondent 39: *“Need more breastfeeding support, places where mums can actually stay overnight or for several days as an inpatient with baby”.*

Respondent 15: *“Would be good to have a birth centre where mother can stay overnight in a private room (like in New Zealand). Or stay a couple of nights without being in hospital”.*

Women commented on issues of crowding at hospitals, including long waiting times and lack of seating in antenatal clinics. Lengthy waiting times for antenatal appointments were described as being particularly difficult for women who had other children.

Respondent 105: *“Waiting times for an appointment with midwives was quite long and not enough room for all the pregnant women to sit down and wait. The longest I waited was 2.5 hours”.*

Respondent 33: *“…every time I had an ante-natal appointment, I had to wait at least an hour. … being made to wait so long each time I had an appointment was very tiring, especially since I had to take my 2 year old with me every time”.*

Some women also reported a dearth of beds in birth suites or postnatal wards, leading to dissatisfaction and general discomfort.

Respondent 68: *“When I arrived for my caesarean, there were no beds available, I had to wait in an office with another couple until I had my baby (6.30 am - 1 pm). It was a bit awful, hard to relax…”.*

Respondent 117: *“I was put in a room with 3 other new mothers and their babies. With the tear and to avoid infection, I was advised to have a shower every time I went to the toilet. This was extremely difficult when sharing with 3 other new mothers…”.*

In some instances, overcrowding was reported to limit women’s available options and preferences for their labour and birth.

Respondent 28: *“With initial induction, it worked well. However midwives stopped induction as I was told they had no birthing suites available…. I found out that the previous day 15 of the women in birthing had had their babies, but were still in birthing suites as no beds in wards. Instead of swapping us around my induction was stopped and I had to have an emergency caesarean. This angered me as I felt the options were taken away from my husband and I”.*

## Discussion

This study highlights a number of prominent issues for women birthing in Queensland, and generally supports the relevance of many current state and national maternity care reform plans. The findings identified particular aspects of care that women would like to see enhanced and provide some specific guidance for how reforms may best be enacted to meet women’s preferences. Furthermore, additional issues pertaining to maternity care that are not explicitly addressed in current reform plans were identified.

Concerns most commonly expressed by women were related to the quality of interpersonal interactions with staff (primarily with midwives). Midwives’ lack of empathy and rude or uncaring behaviour were paramount in women’s assessment of their maternity care experience. The salience of interpersonal interactions (e.g. being treated with kindness or empathy) for women’s satisfaction with maternity care and postnatal functioning is well established [23-27]. Intimidation, bullying and discrimination of women by midwives has been reported in previous research [28-31] and our findings confirm these as critical influences on women’s emotional wellbeing and having their needs met (particularly for birth and infant feeding). Women in this study commonly attributed satisfaction to positive experiences in their interpersonal care, alongside high quality information provision. It makes intuitive sense that information exchange would be enhanced when interpersonal factors are favourable, and this has been documented in health care settings in general [32].

The findings of this study suggest that fundamental aspects of respectful interpersonal interaction are not always achieved. However, improving interpersonal care is not articulated as an explicit goal of current reform plans to ensure high-quality care [8]. Respect and kindness are core principles of the Australian Code of Ethics for Midwives [33], and values of respect, dignity and consideration are espoused in the Australian Charter of Healthcare Rights [34]. Nevertheless, current maternity reform plans may need to be adapted or supplemented to facilitate the translation of these values into maternity care improvement planning.

Aspects of the organisational culture midwives experience during their training and professional lives (e.g., bureaucratic requirements, issues of understaffing, and rostering rotations between wards) may hinder their ability to effectively develop rapport with women and provide optimal emotional and practical support [35-38]. Therefore, reform plans to support a sustainable, well-resourced maternity care workforce may somewhat enhance the capacity for improving interpersonal care. Training in effective interpersonal communication needs further specific attention in reform planning, alongside and as per proposed action to ensure cultural competence is a component of all training, education and professional development of the broad maternity care workforce.

Activities proposed in the National Plan to introduce national clinical quality and safety standards and transparent performance monitoring, and to ensure maternity service planning is woman-centred, should better integrate interpersonal aspects of care. Limiting performance standards, indicators, and their routine assessment and reporting to clinical factors alone overlooks a key opportunity to integrate the combined activities of reform plans and respond to women’s priorities for improvement. Mechanisms for monitoring interpersonal aspects of care have been implemented in Queensland since 2010, resulting in consumer evaluation reports that provide detailed information on the interpersonal aspects of care for women in individual birthing facilities [39]. Similar initiatives should be considered at the national level.

While safety is an essential component of care, this goal may be prioritised at the cost of effective interpersonal interaction when external demands on care providers are increased. Although few women reported dissatisfaction with technical or clinical aspects of care, the propensity for aspects of poor interpersonal care (i.e., failure to respond to information) to compromise clinical safety was described. Previous research has demonstrated that women feel a loss of control over their maternity experience when the information they provide is overlooked by staff [40-42]. Disregard for potentially important information can thus both compromise safety and demonstrate a lack of respect for women or other care providers. Queensland and National reform plans highlight the need for greater integration of care across settings [7,8] and action is already underway to facilitate communication between care providers (e.g., introduction of woman-held Pregnancy Records) [8,43]. Strategic reform activities should have a women-centred approach and foster a culture of interdisciplinary collaboration in maternity care. To achieve this, such activities should be designed with adequate consideration of the patient perspective and provide usable strategies for care providers to improve collaborative communication with both women and other care providers as critical people in the care team.

Current maternity care reforms emphasise the importance of women having a choice and being involved in the care they receive as a fundamental aspect of woman-centred care (see Table 1) [44,45]. Approximately one-third of the women in this sample made unprompted reports that their choices in relation to antenatal, birth or postnatal care were compromised. Previous literature has described the difficulties faced by birthing women in negotiating their preferred maternity care (e.g., preferred pain relief options) [29,40]. Women in the current study perceived their choices were hindered by lack of information, limited access to certain options, and negative interpersonal relations with staff, leaving a number of women feeling coerced into decisions or judged for the decisions that they made. Being involved in decision-making about care and having the ability to exercise choices and preferences are prescribed in the Australian Charter of Health Care Rights, [34] and contribute to women’s satisfaction with their maternity care. Specifically, having autonomy and a sense of control during birth has been found to enhance women’s experiences [25,35,46].

The findings of this study reinforce the importance of national maternity reform objectives to facilitate continuity of care [8], a long held goal for maternity care improvement [25]. Continuity of care is desired by Queensland women, was seen as important for allowing them to develop a relationship with their care providers, and described as most beneficial in midwifery-led models. Midwife-led care has been associated with higher levels of satisfaction in the intrapartum period in previous Australian [25] and international research [47]. Continuity of midwifery care has also been shown to be safe and cost-effective relative to standard care for women with all levels of risk [48]. Women in this study expressed concerns over access to this type of service in general, and specifically indicated that women may not be fully aware of their options for selecting a model of care. Our findings suggest that the desire for continuity of midwifery care, and specifically birth centre care, is unmet in existing service provision. In Queensland, current targets for 10% of births to occur in midwifery continuity models by 2013 [49] seem conservative and in need of revision to meet women’s demand. Although the national plan includes action to utilise midwives to their full scope of practice, the accessibility of birth centre care is not specifically highlighted and requires attention.

Reform plans that focus on creating sufficient workforce and maternity care infrastructure for enhanced access to continuity of midwifery care may be especially important for successfully enacting other reform priorities, such as those to enable informed choice. Women see greater continuity of carer as a means of improving interpersonal interactions and consequently enhancing their access to information, findings that are entwined with aforementioned themes, indicating a need for increased attention to interpersonal care improvements.

Consistent with previous research, women in this study expressed concern that they were given contradictory or incorrect information, insufficient information on how to care for their baby and maintain their own health postpartum, and not enough information on breastfeeding specifically [42]. Information provided was contradictory, lacked sufficient detail (e.g. regarding breastfeeding and caring for a newborn) or timing (e.g. when women in rural and remote areas would need to leave their community to attend hospital for birth), or was withheld based on false assumptions (e.g. multiparous women not advised and supported with breastfeeding). These findings indicate that goals of ‘access’ [8], ‘consumer involvement and choice’ and improving postnatal service delivery in current reform plans [7,2] are critical for addressing the primary deficiencies noted by women in this study, and hinge on the provision of adequate and appropriate information. However, existing reform plans to facilitate women’s access to alternative, objective information sources (e.g., National Pregnancy Helpline) are unlikely to minimise the dissatisfaction associated with receiving contradictory information from staff. Further efforts are needed to enable the front-line workforce to deliver the most current, evidence-based information to women and their families. Increasing the accessibility and affordability of breastfeeding support services, greater access to postnatal home visits, postnatal group classes and community drop-in centres were offered by women as potential areas for action. Although access issues for women in regional and remote communities are well addressed in existing maternity reform plans, the specific information needs for this population subgroup requires attention in activities concerning information provision. Women described the stressful nature of planning to travel for birth when they received insufficient information about factors such as when to leave or whether local hospitals could accommodate their needs.

A foremost goal of both Queensland and National maternity care reform is to improve access to maternity care and health outcomes for Aboriginal and Torres Strait Islander Australians (hereafter, respectfully Indigenous Australians), women living in rural and remote areas and other disadvantaged groups (e.g. socioeconomically disadvantaged groups). The analysis undertaken did not reveal specific maternity care access issues pertaining to Indigenous Australians, possibly due to underrepresentation of this group in the study sample. In both the overall HABIQ 2010 study sample, and the sample drawn for this study, Indigenous women represented only 2.0% of all participants (compared with 5.7% of the Queensland birthing population in 2010) [18]. Further, to facilitate the goal of consumer engagement, consultation and improved maternity care services for Indigenous and other disadvantaged groups, these populations need to be specifically engaged to provide input [50].

Current reforms articulate explicit goals to provide safe and high quality care, and our findings offer discrete areas for improvements to the care environment and infrastructure that can facilitate meeting these objectives. Crowding was noted by some women as influencing the quality and safety of their care in terms of personal comfort, access to care and emotional distress. Women described delays in receiving medical procedures or that their birth choices were altered due to insufficient beds or limited access to birthing suites. Many women were concerned and distressed about the incapacity for their partners to stay or rest at the hospital.

### Strengths and limitations

A self-complete survey was deemed an effective way to obtain information from women with young babies. Although this data collection method precluded the researchers being present to observe non-verbal cues, or to probe for additional information relevant to interpreting the data, self-complete surveys can be comparable to interviews in terms of obtaining authentic, rich and detailed qualitative data [51]. The value and limitations of free-text survey items in particular have been critiqued elsewhere [22,52,53]. In brief, open-text questions do not limit the range of topics that respondents may raise [54], however, may generate an unwieldy volume of data to critically analyse [22]. In the current study, manageability of the analysis was maintained by limiting the sample to a random selection of 150 women. Although the four main themes detailed in this paper were prominent, data saturation did not occur, and additional themes (particularly minor themes) may have arisen had a larger sample been considered.

As discussed earlier, care for Indigenous women did not emerge as key theme despite being prominent in both national and Queensland plans for reform. More targeted research in this population and other population subgroups not well represented in this study (e.g., rural/remote and culturally and linguistically diverse women) is required to adequately assess their unmet needs.

The 150 women included in this study appeared characteristically similar to the 61,027 women birthing in Queensland. However, women under 24 years of age and those living outside of urban areas appeared slightly under-represented in this sample which may limit the generalisability of the findings to these women.

It has been posited that patients may inflate their reported satisfaction with medical services due to fear that negative feedback could result in services being lost [55]. It has also been theorised that women may be more inclined to evaluate their maternity care experiences favourably having arguably just survived the challenge of giving birth [25]. With these perspectives in mind, the complaints and suggestions for improvement expressed in this paper may well be a conservative indication of the changes to maternity care desired by Queensland women.

Some initiatives based on these reform objectives were being implemented when the data were being collected and may have influenced women’s care experience in ways that are not reflected in our findings. For example, Medicare rebates for private midwifery (potentially improving access to midwife-led care and continuity of care) were introduced in November 2010. The findings of this study are likely to be influenced by such circumstances. However, they remain as one valuable contribution to future evaluation of the extent to which maternity care reforms were successfully implemented and for interim self-evaluation of how well current activities as part of Queensland and national plans are responding to women’s needs.

## Conclusions

This study demonstrates the complex constellation of factors that may enhance or detract from women’s maternity care experience. The findings highlight the inter-relatedness of reform objectives and the necessity to address multiple elements simultaneously, and with consistency in applying overarching principles across activities, in order to achieve the overall goal of enhancing maternity care. The analysis of maternal-reported qualitative data provided a unique opportunity to evaluate whether current maternity care reform plans are likely to address key concerns of women birthing in Queensland. The techniques used in this research offer useful information for guiding reform efforts, particularly where there is a commitment to consumer-centred processes for setting agendas and priorities for research and improvement.

Overall, the comments of women supported the importance of many planned reforms. This study consolidates and expands previous research by providing tangible suggestions to address particular reform items, and by identifying issues not explicitly addressed in current plans for maternity care reform. Particular attention to facilitating adequate interpersonal aspects of care when implementing activities associated with current plans is likely to maximise the achievement of overarching goals of current maternity system reform efforts.

## Endnote

^a^Women who completed the mail or online version of the survey.
